# The modulation of radiation-induced damage to pig skin by essential fatty acids.

**DOI:** 10.1038/bjc.1993.276

**Published:** 1993-07

**Authors:** J. W. Hopewell, M. E. Robbins, G. J. van den Aardweg, G. M. Morris, G. A. Ross, E. Whitehouse, D. F. Horrobin, C. A. Scott

**Affiliations:** Research Institute (University of Oxford), Churchill Hospital, UK.

## Abstract

The ability of essential fatty acids (EFAs) to modulate radiation-induced normal tissue injury was assessed in pig skin. Female Large White pigs (approximately 25 Kg) received 3 ml/day orally of either an 'active' oil [So-1100, containing 9% gamma-linolenic acid (GLA)] or a 'placebo' oil (So-1129) for just 4 weeks before or for 4 weeks before and for 16 weeks after irradiation; localised irradiation of skin was with single doses of beta-rays from 22.5 mm diameter 90Sr/90Y plaques. The severity of the acute reaction, assessed in terms of erythema or moist desquamation, was significantly less in those pigs that received So-1100 both before and after irradiation, as compared with those receiving that oil only prior to irradiation and the 'placebo' groups. Dose modification factors (DMFs) of between 1.13-1.24 were obtained. A similar reduction in the severity of acute skin injury was seen in pigs receiving So-1100 for only 10 weeks after irradiation. Late skin damage, assessed in terms of late erythema or dermal necrosis, was also reduced with So-1100, with DMFs of 1.14-1.51. No such modification was observed if So-1100 was only administered for 4 weeks prior to irradiation. No adverse side-effects were apparent as a result of EFA administration. So-1100 may represent a safe and valuable method of increasing the therapeutic gain in radiotherapy.


*Br. J. Cancer* (1993), **68**, 1–7 © Macmillan Press Ltd., 1993

### The modulation of radiation-induced damage to pig skin by essential fatty acids

J.W. Hopewell[1], M.E.C. Robbins[1], G.J.M.J. van den Aardweg[1], G.M. Morris[1], G.A. Ross[1], E. Whitehouse[1], D.F. Horrobin[2] & C.A. Scott[2]

[1]*CRC Normal Tissue Radiobiology Research Group, Research Institute (University of Oxford), Churchill Hospital, Oxford, OX3 7LJ;* [2]*Scotia Pharmaceuticals Ltd., Woodbridge Meadows, Guildford, Surrey, GU1 1BA, UK.*

**Summary** The ability of essential fatty acids (EFAs) to modulate radiation-induced normal tissue injury was assessed in pig skin. Female Large White pigs (~25 Kg) received 3 ml/day orally of either an 'active' oil [So-1100, containing 9% gamma-linolenic acid (GLA)] or a 'placebo' oil (So-1129) for just 4 weeks before or for 4 weeks before and for 16 weeks after irradiation; localised irradiation of skin was with single doses of $\beta$-rays from 22.5 mm diameter $^{90}Sr/^{90}Y$ plaques. The severity of the acute reaction, assessed in terms of erythema or moist desquamation, was significantly less in those pigs that received So-1100 both before and after irradiation, as compared with those receiving that oil only prior to irradiation and the 'placebo' groups. Dose modification factors (DMFs) of between 1.13–1.24 were obtained. A similar reduction in the severity of acute skin injury was seen in pigs receiving So-1100 for only 10 weeks after irradiation. Late skin damage, assessed in terms of late erythema or dermal necrosis, was also reduced with So-1100, with DMFs of 1.14–1.51. No such modification was observed if So-1100 was only administered for 4 weeks prior to irradiation. No adverse side-effects were apparent as a result of EFA administration. So-1100 may represent a safe and valuable method of increasing the therapeutic gain in radiotherapy.

In radiotherapy the dose that can be administered to a tumour is limited by the risk of early or late morbidity to those normal tissues included within the treatment volume. Several approaches, in addition to modifications in fractionation schedules, have been used in an attempt to improve the therapeutic ratio. In general, these have focused on, either the enhancement of tumour cell radiosensitivity by overcoming the problem of hypoxia, using sensitisers, or the selective protection of normal tissues using radioprotectors. Such approaches have achieved limited success (Overgaard *et al.*, 1992; Dische, 1992) and have often been hampered by problems of agent-associated toxicity (Blumberg *et al.*, 1982; Overgaard *et al.*, 1989).

An alternative approach is to utilise interventional procedures after radiation exposure, which are directed at selectively reducing the severity of normal tissue morbidity without comprising tumour cell kill. Such as approach, using various biological response modulators (BRMs), could bring significant clinical benefits. Recent studies wtih various agents, which might be termed BRMs, have demonstrated significant amelioration of radiation-induced normal tissue morbidity. These include the use of Captopril an angiotensin converting enzyme inhibitor (ACE), effective in reducing radiation damage to skin (Ward *et al.*, 1990), lung (Ward *et al.*, 1992) and kidney (Robbins & Hopewell, 1986); Dipyrimidazole and Desferrioxamine in the spinal cord (Hornsey *et al.*, 1990), and Pentoxifylline (PTX) in cutaneous tissue (Dion *et al.*, 1989). PTX has been shown to be of significant clinical benefit in the treatment of late-induced radiation soft tissue necrosis (Dion *et al.*, 1990).

The precise mode of action of individual BRMs remains uncertain, however, there is a growing realisation that the development of radiation-induced normal tissue morbidity reflects a cascade of complex events in which both direct and indirect effects of radiation on cells results, ultimately, in the expression of organ damage. These events include alterations in eicosanoid metabolism. *In vitro* and *in vivo* studies of radiation effects on the vasculature indicate an initial increase in prostacyclin ($PGI_2$) production followed by a long-term reduction; this can last for $\geqslant 12$ months (Sinzinger *et al.*, 1984; Eldor *et al.*, 1989; Rubin *et al.*, 1991). Thromboxane ($TXA_2$) production appears to be unaffected (Allen *et al.*, 1981). A similar imbalance in the ratio of these two dienoic eicosanoids has been reported *in vivo* following irradiation of the kidney (Schneidkraut *et al.*, 1984; Weshler *et al.*, 1987) and the lung (Ward *et al.*, 1990). Moreover, increases in prostaglandins (PGs) and inflammatory mediators, (leukotrienes-LTs), have also been reported in the gut and oral mucosa (Abdelaal *et al.*, 1989; Cole *et al.*, 1993) after irradiation.

Although there is a tendency to assume that the EFAs are important only as precursors of eicosanoids, their most important role is related to the structure of membranes, where they regulate membrane fluidity and flexibility and the functions of membrane related proteins such as receptors, ion channels, ATPases and the proteins associated with secondary messenger systems (Horrobin, 1992). Therefore, it is possible that the loss of EFAs themselves may be a major factor in the development of radiation-induced damage to normal tissues (Horrobin, 1991; Stark, 1991).

The above findings suggest that the prevention of alterations in eicosanoid and EFA metabolism could lead to a reduction in normal tissue morbidity. Indeed, the direct use of exogenous PGs and/or inhibitors of PG production, such as indomethacin, have proved beneficial (Tochner *et al.*, 1990). However, an alternative approach appears to be the modification of essential fatty acid (EFA) levels, the precursors of eicosanoids (Willis, 1981). Administration of gamma-linolenic acid (GLA) has been shown to result in an increased production of the monoenoic $PGE_1$ rather than dienoic PGs such as $PGE_2$ and $TXA_2$. Moreover, GLA itself leads to the formation of 15-OH-dihomogammalinolenic acid, an inhibitor of LT formation (Horrobin & Manku, 1990). $PGE_1$ has a number of desirable physiological actions (Zurier, 1990); these include anti-inflammatory, anti-aggregatory, and vasodilatory activity.

Although the main essential fatty acid in the diet is linoleic acid, the most important constituents of all membranes are the linoleic acid metabolites dihomogammalinolenic acid (DGLA) and arachidonic acid (AA). Conversion of linoleic acid to its first metabolite, GLA, is slow and rate limiting, especially in humans. In the present study the properties of an oil containing linoleic acid (So-1129) with an oil contain-

Correspondence: J.W. Hopewell.
Received 6 November 1992; and in revised form 5 February 1993.

ing linoleic acid and GLA (So-1100) are compared. The difference between the two oils allows an evaluation of the role of EFA metabolites in modulating radiation-induced damage.

To assess the ability of EFAs to ameliorate radiation-induced normal tissue injury the effects of So-1100 on acute and late radiation responses in pig skin were assessed. These skin responses were compared with those observed in pigs receiving a 'placebo' oil So-1129 (oil containing 79% LA and no GLA). In order to resolve whether GLA might act as a radioprotector rather than a BRM these oils were given either up to the time of irradiation only or both before and after irradiation. PGs have been reported to protect against radiation damage (Hanson & Ainsworth, 1985). EFA administration was initiated 4 weeks prior to irradiation to ensure stable EFA levels in the animals before local skin irradiation. The pig is the ideal laboratory animal for such studies, as pig and human skin are essentially identical in terms of their structure and response to radiation (Hopewell, 1986). Furthermore, the assay systems used produce highly reproducible data (Hopewell & van den Aardweg, 1988).

### Materials and methods

A total of 16 female pigs of the Large White strain were used in these investigations. Animals were approximately 12 weeks of age (20–25 kg) when they were brought into the animal house and were allowed an acclimatisation period of 2 weeks prior to any experimental procedures being carried out. A group of 12 pigs then received oils orally, 3 ml/day for 4 weeks. Administration of oil was via a syringe so that the oil could be directed towards the back of the oral cavity. These animals were randomly allocated to either what was termed an 'active' oil (So-1100, containing 9% GLA) or to a 'placebo' oil (So-1129, Sunflower oil). The main difference between these two oils is that there is 9% GLA in So-1100 and no GLA in So-1129. Otherwise the fatty acid compositions are not dissimilar (Table I). The remaining four pigs received no oils over this period.

Three weeks from the start of administration of the oils and 1 week prior to irradiation the skin sites to be irradiated, 16 per flank, were marked out by tattooing with India ink. These sites were 25 mm in diameter with 40 mm between sites. Subsequent irradiation was with single doses of $\beta$-rays from 22.5 diameter $^{90}Sr/^{90}Y$ plaques (Amersham International, UK). Skin surface doses (measured at 16 $\mu$m depth) were given at a rate of $\sim$3.0 Gy/min. For animals given the 'active' oil, total doses in the range 20–40 Gy were used; doses of 20–34 Gy were used for the 'placebo' group. In each treatment group 5 different radiation dose levels were used; 10–12 sites were irradiated to each dose at randomly selected skin fields. For all the experimental procedures the pigs were anaesthetised with a mixture of $\sim$70% oxygen, $\sim$30% nitrous oxide and 2% halothane (Dickinson & Hubbard, 1990).

At the time of irradiation daily administration of oils was discontinued in four of the 12 pigs (two 'active'; two 'placebo'), the remaining eight pigs (six 'active'; two 'placebo') continued to receive oil at 3 ml/day for a further 16 weeks. The four pigs that had received no oil prior to irradiation, were given it at the same dose (two 'active'; two 'placebo') for a period of 10 weeks after irradiation.

To assess the severity of the early skin reactions the irradiated skin sites were examined at weekly intervals for 10 weeks by at least 3 observers. Erythema was evaluated as being either minimal (A), moderate (B) or bright red (C) and the sites were also assessed as to the presence or absence of moist desquamation (van den Aardweg et al., 1988). Dose-related changes in the skin response were initially evaluated on the basis of the ordinal data for erythema by calculating, for each dose level, an average erythema score over the period 3–9 weeks after irradiation. For this purpose an arbitrary numerical score was assigned to the different visual observations (Hopewell et al., 1978) and dose-effect curves fitted by linear regression analysis. Different treatment schedules were compared on the basis of an average score of 1.5 obtained from the dose-effect curves. Alternatively, responses were converted into quantal data by assessing the dose-related changes in the percentage of skin sites that showed, over the time course of the acute reaction: (a) $\geqslant$ moderate erythema; (b): bright red erythema; or (c) moist desquamation. These quantal data were analysed using probit analysis and $ED_{50}$ ($\pm$ s.e.) values calculated in order to compare different treatment schedules.

In the eight pigs that received oil (six 'active'; two 'placebo') for a period up to 16 weeks after irradiation, and the four animals that only received oils (two 'active'; two 'placebo') up until the time or irradiation, the skin reactions were assessed for a further period of 6 weeks (weeks 10–16). The severity of the later dermal reaction in these animals was compared on the basis of the dose-related incidence of dusky/mauve erythema and of dermal necrosis (van den Aardweg et al., 1988).

### Results

Daily oral administration of oils (both the 'active' and 'placebo') was achieved without difficulty, the animals accepting these prior to normal feeding early each morning. Administration of the 'active' oil, even over a prolonged period of 20 weeks, appeared to have no adverse effects on the general condition of the animals. The increase in weight of the two pigs which received the 'placebo' oil for a full 20 weeks was within the same range as those receiving the 'active' agent (Figure 1).

**Figure 1** Time-related changes in the body weight ($\pm$ s.d.) of pigs receiving the 'active' oil, So-1100 (●—●) for 4 weeks prior to and for 16 weeks after local skin irradiation. The individual body weight values for the two pigs receiving the 'placebo' oil, So-1129, (Δ,▲) are given for comparison.

**Table I** Composition of the fatty acids used in the studies described in this paper

| | *So-1129* | *So-1100* |
|---|---|---|
| Linoleic (18:2*n*-6) | 79.0 | 70.2 |
| Gamma-linolenic (18:3*n*-6) | – | 8.7 |
| Oleic (18:1*n*-9) | 10.7 | 12.7 |
| Stearic (18:0) | 2.1 | 1.8 |
| Palmitic (16:0) | 6.9 | 5.8 |
| Alpha-linolenic (18:3*n*-3) | 0.6 | – |
| Others | 0.7 | 0.8 |

The dose-related changes in the average erythema scores, obtained 3–9 weeks after irradiation, for pigs that only received oils for the 4 weeks prior to irradiation are shown in Figure 2a. Separate linear-dose-response curves have been fitted to the data for the 'active' (So-1100) and the 'placebo' (So-1129) oil. For doses in the range 20–40 Gy, average erythema scores varied from 1.2–2.2 arbitrary units. Based on an average erythema score of 1.5, iso-effect doses of 21.4 ± 1.4 Gy and 26.5 ± 1.6 Gy were obtained for the 'active' and 'placebo' groups, respectively. This difference was statistically significant ($P<0.02$) suggesting a slight enhancement of the radiation response by the 'active' oil [dose modification factor (DMF) = 0.81 ± 0.07].

A similar set of data for four pigs receiving oils both prior to and after irradiation are illustrated in Figure 2b. These animals were irradiated at the same time and their reactions assessed over the same time period as those animals the results for which are shown in Figure 2a. The most obvious feature is that the average erythema scores are lower (range 0.6–1.6) in those pigs receiving oils after irradiation and over the time course of the radiation response. The iso-effect doses based on an average erythema score of 1.5, were 37.1 ± 2.4 Gy and 30.7 ± 2.2 Gy for the 'active' and 'placebo' groups, respectively. These iso-effect doses were significantly different ($P<0.05$). This suggested a DMF of 1.21 ± 0.12. The higher iso-effect dose obtained for the 'placebo' group when the oil was given both before and after irradiation (30.7 ± 2.2 Gy) as compared with only prior to irradiation (26.5 ± 1.0 Gy) was not statistically significant ($P>0.05$). However, the degree of modification in the radiation response on the above plots appears to be a function of the severity of the reaction and is also subject to the assumption that the relationship between average skin score, based on an arbitrary scale, and dose is linear.

In view of these concerns, these data and the observations from subsequent animals were transformed into quantal data in order to establish dose-effect relationships for ≥ moderate erythema (≥ B) and bright red (C) erythema. A skin site was adjudged to represent a responder if more than half of the observers assessed the reaction as being either ≥ B or C in any single week over the period of the early reaction.

The $ED_{50}$ values for the different severities of erythema for the various treatment groups are listed in Table II. An illustrative dose-effect curve for the incidence of bright red erythema is shown in Figure 3. In this figure data for the two 'placebo' groups, i.e. So-1129 given either only prior to irradiation or before and after irradiation, have been combined, since the radiation response of the two groups was similar. The $ED_{50}$ values (± s.e.) were 29.68 ± 1.68 Gy and 31.76 ± 1.39 Gy, respectively ($P>0.45$). A similar conclusion was reached on the basis of comparing the $ED_{50}$ values for ≥ B grade erythema. However, the $ED_{50}$ values for ≥ B and C grade erythema for the group of pigs receiving So-1129 both before and after irradiation, was higher (~11%) than that for those receiving this oil only prior to irradiation. This trend was again repeated in the group that only received So-1129 after irradiation; in this case the difference in the $ED_{50}$ values for C grade erythema approached statistical significance ($P<0.1>0.05$).

The dose-effect curves for the incidence of bright red erythema (Figure 3) illustrate the marked difference in response observed when So-1100 was given both before and after irradiation as compared with before irradiation only. The response following administration of So-1129, the placebo, was somewhat intermediate between the two. When $ED_{50}$ values for both C and ≥ B grade erythema after So-1100 administration were compared with the associated results for the 'placebo' group a dose modification factor of ≈1.2 ($P<0.005$) was obtained for the before and after irradiation group. No significant change in response was seen when So-1100 was given only prior to irradiation ($P>0.1$) (Table II). The suggestion that to be effective So-1100 has to be given over the time course of the early radiation response is supported by the results from the group of pigs that received oils only after irradiation. A dose modification factor of 1.2 ± 0.14 was obtained for the incidence of a C grade erythema ($P<0.1>0.05$) in these studies. No significant modification was noted based on the end point of a ≥ B

**Figure 2** Dose-related changes in the erythema scores in pig skin averaged over the period 3–9 weeks after irradiation with single doses of $^{90}Sr/^{90}Y$ β-rays. Results are for animals receiving EFAs either for 4 weeks prior to irradiation **a**, or for 4 weeks prior to irradiation and 16 weeks after irradiation **b**. Animals received either the 'active' oil, So-1100 (O), or the 'placebo' oil, So-1129 (●). Error bars indicate ± s.e.

**Table II** Variation in $ED_{50}$ values for different severities of erythema in pig skin after irradiation with single doses of $^{90}Sr/^{90}Y$ β-rays and pre- and post-irradiation treatment with oils (So-1100 and So-1129)

| *Treatment period (weeks)* | *Severity of reaction* | *$ED_{50}$ ± s.e. (Gy)* So-1100 | So-1129 | *DMF* |
|---|---|---|---|---|
| −4 | C | 26.81 ± 1.15 | 29.68 ± 1.68 | NS |
| | ⩾B | <<20 | 20.35 ± 1.29 | – |
| −4/+16 | C | 39.23 ± 0.98 | 31.76 ± 1.39 | 1.24 ± 0.06 |
| | ⩾B | 26.46 ± 0.79 | 22.66 ± 0.69 | 1.17 ± 0.05 |
| +10 | C | 41.16 ± 3.79 | 34.44 ± 2.35 | 1.2 ± 0.14 |
| | ⩾B | 23.34 ± 2.28 | 22.49 ± 1.96 | NS |

NS = no significant dose modification

**Figure 3** Dose-related changes in the incidence of bright red erythema in pig skin after irradiation with single doses of $^{90}Sr/^{90}Y$ β-rays. Animals received an 'active' oil, So-1100, for either 4 weeks prior (□) or for 4 weeks prior and 16 weeks after irradiation (○) or a 'placebo' oil, So-1129 both before and after irradiation (●). $ED_{50}$ values (± s.e.) are indicated.

grade erythema. However, the data from this group of animals showed considerably greater scatter than those from other groups. Based on the results for the incidence of C grade erythema, the degree of modification produced by So-1100 given both before and after irradiation was similar to that seen when it was only given after irradiation.

The results obtained based on the endpoint of moist desquamation are shown in Table III. In the pigs receiving oils for only 4 weeks prior to irradiation the $ED_{50}$ values were both ~27 Gy, not significantly different from historical data for pig skin when irradiation was carried out without the administration of oils (Hopewell & van den Aardweg, 1988). The administration of oils, both 'active' and 'placebo', over the time course of the radiation reaction resulted in higher $ED_{50}$ values for moist desquamation. They were significantly higher ($P < 0.05$) except for the 'placebo' group (−4/+16 weeks). In the case of the groups receiving oils both prior to and after irradiation the difference in $ED_{50}$ values for moist desquamation between the 'active' and 'placebo' was highly significant ($P < 0.005$) suggesting a DMF of 1.13 ± 0.05.

**Table III** Iso-effect doses ($ED_{50}$) for moist desquamation in pig skin after pre- or post-irradiation treatment with So-1100 ('active') or So-1129 ('placebo') oils

| *Treatment period (weeks)* | *$ED_{50}$ ± s.e (Gy)* So-1100 | So-1129 | *DMF* |
|---|---|---|---|
| −4 | 26.00 ± 1.87 | 27.91 ± 1.15 | NS |
| −4/+16 | 33.81 ± 0.8 | 30.04 ± 1.18 | 1.13 ± 0.05 |
| +10 | 31.74 ± 1.08 | 31.03 ± 1.42 | NS |

Historical control $ED_{50}$ (± s.e.) values for moist desquamation 27.32 ± 0.5 Gy (Hopewell & van den Aardweg, 1988).

The modification in the severity of the early skin reaction of moist desquamation, and the possible effect of the 'placebo' agent when given over the time course of the early skin reaction, was supported by the observations on the duration of moist desquamation for specific dose levels above the $ED_{50}$ (Table IVa). For single doses in the range 28–32 Gy the healing times for moist desquamation were in the order of 2.7–3.7 weeks when the oils were only given prior to irradiation. Extending the administration of the oils over the period associated with the development of moist desquamation reduced this to 2.2 weeks for the 'placebo' group and to only 1.2 weeks for the 'active' oil. However, the time of onset of moist desquamation was not influenced by the continued administration of oils over the period of the early reaction (Table IVb).

For the assessment of late, vascular mediated, lesions in pig skin the incidence of a second wave of dusky/mauve erythema and ischaemic necrosis was assessed 10–16 weeks after irradiation. The $ED_{50}$ values for these two dermal reactions are given in Table V. Prior treatment with So-1100 did not significantly modify the responses with respect to those seen after a similar treatment with So-1129. The administration of So-1129 over the time course of the reaction also produced no further changes in the severity of the reaction. However, administration of So-1100 over the time course of the late dermal reaction significantly reduced its severity; DMFs of 1.14 ± 0.06 and 1.51 ± 0.12 were obtained for necrosis and dusky/mauve erythema, respectively.

### Discussion

The present findings clearly demonstrate that the administration of EFA metabolites had a significant effect on both the acute and late radiation responses of pig skin. The nature of these effects was dependent on the particular EFA dose regimen used. Thus in animals that received So-1100 or the 'placebo' So-1129 for 4 weeks up to the time of irradiation alone there was no evidence of any reduction in the severity of the radiation-induced skin damage. Indeed, analysis of

**Table IV** Time of onset and duration of moist desquamation in pig skin irradiation with single doses of $^{90}Sr/^{90}Y$ β-rays

| *Treatment (weeks)* | *Dose (Gy)* | *So-1100* | *Dose (Gy)* | *So-1129* |
|---|---|---|---|---|
| **(A)** Duration moist desquamation (weeks; mean ± s.e) | | | | |
| −4 | 28 | 2.7 ± 1.1 | 29 | 3.4 ± 1.0 |
| | 32 | 3.1 ± 0.7 | 31 | 3.7 ± 0.7 |
| −4/+16 | 28 | 1.3 ± 0.3 | 29 | 2.2 ± 0.6 |
| | 32 | 1.2 ± 0.2 | 31 | 2.2 ± 0.7 |
| **(B)** Onset of moist desquamation (weeks; mean ± s.e) | | | | |
| −4 | 28 | 4.7 ± 0.2 | 29 | 5.2 ± 0.5 |
| | 32 | 4.4 ± 0.3 | 31 | 4.5 ± 0.3 |
| −4/+16 | 28 | 5.0 ± 0.6 | 29 | 5.2 ± 0.4 |
| | 32 | 5.4 ± 0.6 | 31 | 5.4 ± 0.7 |

**Table V** Iso-effect doses ($ED_{50}$) for both dermal necrosis (N) or dusky/mauve erythema (E) in pig skin after pre- and post-irradiation treatments with So-1100 ('active') or So-1129 ('placebo') oils

| *Treatment period (weeks)* | *Type of reaction* | *$ED_{50} \pm$ s.e. (Gy)* *So-1100* | *So-1129* | *DMF* |
|---|---|---|---|---|
| −4 | N | 34.8 ± 1.4 | 35.0 ± 1.5 | NS |
| | E | 26.5 ± 1.3 | 27.5 ± 1.1 | NS |
| −4/+16 | N | 40.6 ± 1.3 | 35.7 ± 1.6 | 1.14 ± 0.06 |
| | E | 37.5 ± 1.9 | 24.8 ± 1.5 | 1.51 ± 0.12 |

average erythema scores inferred a slight enhancement of the acute skin response in those pigs receiving So-1100. However, no such enhancement was evident for either acute radiation damage, assessed in terms of the incidence of moist desquamation, or for the later radiation damage to dermal tissues, assessed in terms of the incidence of dusky mauve erythema or dermal necrosis. In contrast, administration of So-1100 from 4 weeks prior to irradiation and for the following 16 weeks after irradiation resulted in a significant reduction in the severity of skin damage compared with that seen in pigs which received So-1129 over a similar time-course. DMF values in the order of 1.2 and 1.13 were obtained for the early responses of bright-red erythema and moist desquamation, respectively. These findings indicate that So-1100 significantly reduces the severity of acute radiation-induced morbidity in pig skin when administered over the time-course of the reaction. A similar trend was noted in pigs receiving So-1100 for 10 weeks after irradiation alone. It should be noted that administering the 'placebo' oil So-1129 before and after irradiation did in itself increase the $ED_{50}$ value for moist desquamation compared with that seen in historical control animals (Hopewell & Aardweg, 1988). Thus comparing the $ED_{50}$ value for moist desquamation for pigs receiving So-1100 both before and after irradiation with that from historical control animals resulted in a significantly increased DMF value of $1.24 \pm 0.04$ ($P < 0.001$), an indication as to some effect of the 'placebo' oil, So-1129.

The reduction in the severity of moist desquamation produced by So-1100 was also evident in terms of a reduction in the duration, but not the time of onset, of moist desquamation. The healing times for moist desquamation of some 3–4 weeks observed in the pigs receiving EFAs prior to irradiation alone were reduced in pigs receiving So-1100 or So-1129 both before and after irradiation to approximately 1 and 2 weeks, respectively. The mechanism(s) responsible for this enhanced healing response remains unclear. It may reflect, at least in part, an induced acceleration of epithelial cell turnover by EFAs. Preliminary observations from biopsies of skin taken from pigs receiving EFAs have shown a marked increase in the labelling index of basal cells and an increase in the number of viable cell layers, indicating an increase in cell proliferation kinetics (Morris *et al.*, unpublished data). This could occur via an EFA-induced increase in $PGE_1$ production; exogenous $PGE_1$ has been shown to accelerate gastrointestinal repair by increasing cell proliferation rates (Levi *et al.*, 1990). It may also reflect a direct action of EFAs which are required for membrane synthesis in proliferating cells.

The administration of So-1100 before and after irradiation also resulted in a significant reduction in the severity of late vascular mediated lesions in pig skin i.e. dusky/mauve erythema and dermal necrosis. As seen for the acute reactions, the modification in the response was greater for the erythematous reaction than for necrosis; the DMFs were approximately 1.5 and 1.2, respectively. No modification of late damage was evident in animals receiving EFAs only prior to irradiation, or in those receiving So-1129 both before and after irradiation. The greater modification of the erythematous reaction than the more severe reactions of moist desquamation and dermal necrosis is of interest and suggests a greater ability of GLA to modify the inflammatory aspect of these lesions, possibly through changing membrane structure or by increased production of $PGE_1$ and/or reducing LT production (Horrobin & Manku, 1990). However, the precise mode of action remains to be defined. In particular, measurements of possible alterations in EFA, PG and LT levels occurring as a result of the irradiation of pig skin are required.

It is important to note the lack of any modifying effect seen in animals only treated with So-1100 prior to irradiation, indicating that So-1100 afforded no direct radioprotection. Rather it acts as a BRM in the sense that its presence is required throughout the time-course of the expression of early and late damage. The BRMs Captopril and PTX have also been shown to reduce the severity of late radiation damage to skin (Ward *et al.*, 1990; Dion *et al.*, 1989). However, only Captopril appeared to also reduce the severity of the acute skin response.

The finding that administration of the 'placebo' oil So-1129 also resulted in some amelioration of the acute radiation-induced skin damage is of interest. It should be noted that there is little evidence to suggest that So-1129 is a truly inert 'placebo'. The main EFA constituent of So-1129 is linoleic acid (LA); So-1129 contains 79% LA, while So-1100 contains 70% LA plus 9% GLA (Table I). Administration of LA to normal humans results in increased levels of both LA and arachidonic acid (AA), the precursor of the dienoic PGs (Horrobin *et al.*, 1991). Such an elevation of blood concentrations of the potentially pro-inflammatory AA could worsen clinical disorders in which inflammation, platelet aggregation and vasoconstriction are important, *viz* radiation-induced normal tissue injury. Indeed, administration of So-1129 to children with atopic eczema did worsen the clinical status of these patients. However, no such increase in severity of the acute radiation damage was seen in the pigs receiving So-1129. There is little information yet available concerning EFA metabolism in the pig; in particular, the effect of LA or GLA administration on the blood levels of LA, GLA or AA has yet to be determined.

The DMFs of between 1.13 to 1.5 for acute and late skin reactions represent a potentially clinically significant effect, with in excess of 10% or more dose being required to produce the same level of normal tissue injury. Such a dose increment would, for many tumours, provide a significant improvement in local control by radiotherapy. An important aspect of using such BRMs is that EFAs can be easily administered and appear to be non-toxic. In the present study there were no apparent adverse side-effects associated with the administration of EFAs over periods of up to 20 weeks. Animal toxicology studies using doses up to 5 ml or 10 ml kg/day have also reported no toxic effects attributable to EFA administration (Everett *et al.*, 1988*a*; 1988*b*). Moreover, clinical trials involving the daily administration of very high doses of GLA for periods up to 1 year have revealed no significant adverse side-effects (Dodge, 1990).

Thus administration of EFAs appears to offer a potentially safe way of reducing the severity of radiation-induced normal tissue injury. Recent studies examining the effects of EFAs on tumour cells would seem to indicate that this reduction in radiation-induced injury is specific for normal tissues. GLA administration to co-cultures of normal and malignant cells from the same tissue results in the killing of malignant cells within 5–10 days of exposure; normal cells remain unaffected (Fujiwara *et al.*, 1984; Begin *et al.*, 1986*a*; 1986*b*). Furthermore, *in vivo* studies have shown that GLA inhibits the growth of dimethylbenzanthracene-induced mammary tumours in rats (Lee & Sugano, 1986). This GLA-mediated tumour cytotoxicity may reflect increased lipid peroxidation resulting in the death of tumour cells (Horrobin, 1990). However, the effects of So-1100 and So-1129 on tumour regrowth delay after irradiation have still to be established although such investigations are now underway (Stratford, personal communication). Thus it would appear that the use of EFAs, acting as BRMs, may result in a significant increase in the therapeutic gain in the treatment of cancer by radiotherapy.

The authors would like to thank Mr F. Dickinson and Mr N. Hubbard for their expertise in anaesthesia and for the day-to-day care of the animals and to other members of the Research Institute staff for their help in assessing the skin reactions on pigs. The Research Group at the Churchill Hospital is supported by a Grant from the Cancer Research Campaign.

### References

AARDWEG, VAN DEN, G.J.M.J., HOPEWELL, J.H. & SIMMONDS, R.H. (1988). Repair and recovery in the epithelium and vascular connective tissue of pig skin after irradiation. *Radiother. Oncol.*, **11**, 73–82.

ABDELAAL, A.S., BARKER, D.S. & FERGUSSON, M.M. (1989). Treatment for radiation-induced mucositis. *Lancet*, **i**, 97.

ALLEN, J.B., SAGERMAN, R.H. & STUART, M.J. (1981). Irradiation decreases vascular prostacyclin formation with no concomitant effect on platelet thromboxane production. *Lancet*, **ii**, 1193–1196.

BEGIN, M.E., ELLS, G. & DAS, U.N. (1986*a*). Selected fatty acids as possible intermediates for selective cytotoxic activity of anticancer agents involving oxygen radicals. *Anticancer Res.*, **6**, 291–296.

BEGIN, M.E., ELLS, G., DAS, U.N. & HORROBIN, D.F. (1986*b*). Differential killing of human carcinoma cells supplemented with n-3 and n-6 polyunsaturated fatty acids. *J. Natl Cancer Inst.*, **77**, 1053–1062.

BLUMBERG, A.L., NELSON, D.F., GRAMKOWSKI, M., GLOVER, D.J., GLICK, J.H., YUHAS, J. & KLIGERMAN, M.M. (1982). Clinical trials of WR2721 with radiation therapy. *Int. J. Radiat. Oncol. Biol. Phys*,. **8**, 561–563.

COLE, A.T., SLATER, K., SOKAL, M., FILIPOWICZ, B., KURLAK, L. & HAWKEY, C.J. (1993). Elevated rectal leukotriene $B_4$, Thromboxane $B_2$ and Prostaglandin $E_2$ levels in patients having pelvic radiotherapy. In *Eicosanoids and Other Bioactive lipids in Cancer, Inflammation and Radiation Injury*. Nigam, S., Honn, K.V., Marnett, L.J., Walden, Jr., T.L., (eds) pp. 771–773. Kluwer Academic Publishers, Boston.

DICKINSON, F. & HUBBARD, N. (1990). The Large White female pig in research related to cancer treatment: general husbandry and anaesthesia. *Anim. Technol.*, **41**, 35–41.

DION, M.W., HUSSEY, D.H. & OSBORNE, J.W. (1989). The effect of pentoxifylline on early and late radiation injury following fractionated irradiation in C3H mice. *Int. J. Radiat. Oncol. Biol. Phys.*, **17**, 101–107.

DION, M.W., HUSSEY, D.H., DOORNBOS, J.F., VIGLIOTTI, A.P., WEN, B.C. & ANDERSON, B. (1990). Preliminary results of a pilot study of pentoxifylline in the treatment of late radiation soft tissue necrosis. *Int. J. Radiat. Oncol. Biol. Phys.*, **19**, 401–407.

DISCHE, S. (1992). Radiotherapy, carcinoma of cervix and the radiosensitizer Ro03-8799 (pimonidazole). In *Radiation Research, A Twentieth Century Perspective*, Dewey, W.C., Edington, M., Fry, R.J.M., Hall, E.J. & Whitmore, G.F. (eds.) pp. 584–589, Academic Press; London.

DODGE, J.A. (1990). Essential fatty acids in cystic fibrosis. In *Omega-6 Essential Fatty Acids: Pathophysiology and Roles in Clinical Medicine*, Horrobin, D.F. (ed). pp. 427–435, Wiley-Liss, New York.

ELDOR, A., VLADOVSKY, L., FUKS, Z., MATZNER, Y. & RUBIN, D.B. (1989). Arachidonic metabolism and radiation toxicity in cultures of vascular endothelial cells. *Prostaglan. Leuk. Essential Fatty Acids*, **36**, 251–258.

EVERETT, D.J., GREENOUGH, R.J., PERRY, C.J., McDONALD, P. & BAYLISS, P. (1988*a*). Chronic toxicity studies of Efamol evening primrose oil in rats and dogs. *Med. Res. Sci.*, **16**, 863–864.

EVERETT, D.J., PERRY, C.J. & BAYLISS, P. (1988*b*). Carcinogenicity studies of Efamol evening primrose oil in rats and mice. *Med. Res. Sci.*, **16**, 865–866.

FUJIWARA, F., TODO, S. & IMASHUKU, S. (1984). Antitumour effect of gamma-linolenic acid on cultures human neuroblastoma cells. *Prostaglandins Leukot. Med.*, **15**, 15–34.

HANSON, W.R. & AINSWORTH, E.J. (1985). 16,16-dimethyl prostaglandin $E_2$ induces radioprotection in murine intestinal and hematopoietic cells. *Radiat. Res.*, **103**, 196–203.

HOPEWELL, J.W. (1986). Mechanisms of the action of radiation on skin and underlying tissues. *Br. J. Radiol.* **(Suppl 19)**, 39–47.

HOPEWELL, J.W. & VAN DEN AARDWEG, G.J.M.J. (1988). Radiobiological studies with pig skin. *Int. J. Radiat. Oncol. Biol. Phys.*, **14**, 1047–1050.

HOPEWELL, J.W., FOSTER, J.L., GUNN, Y., MOUSTAFA, H.F., PATTERSON, T.J.S., WIERNIK, G. & YOUNG, C.M.A. (1978). The role of vascular damage in the development of late radiation effects in the skin. In *Late Biological Effects of Ionising Radiation*, I.A.E.A. Vienna, **1**, 483–492.

HORNSEY, S., MYERS, R. & JENKINSON, T. (1990). The reduction of radiation damage to the spinal cord by post-irradiation administration of vasoactive drugs. *Int. J. Radiat. Oncol. Biol. Phys.*, **18**, 1437–1442.

HORROBIN, D.F. (1990). Essential fatty acids, lipid peroxidation, and cancer. In *Omega-6 Essential Fatty Acids: Pathophysiology and Roles in Clinical Medicine*. Horrobin, D.F. (ed) pp. 351–377, Wiley-Liss, New York.

HORROBIN, D.F. (1991). Is the main problem in free radical damage caused by radiation, oxygen and other toxins the loss of membrane essential fatty acids rather than accumulation of toxic materials? *Med. Hypotheses*, **35**, 23–26.

HORROBIN, D.F. (1992). Nutritional and medical importance of gamma-linolenic acid. *Prog. Lipid Res.*, **31**, 163–194.

HORROBIN, D.F., ELLS, K.M., MORSE-FISHER, N. & MANKU, M.S. (1991). The effects of evening primrose oil, safflower oil and paraffin on plasma fatty acid levels in humans: choice of an appropriate placebo for clinical studies on primrose oil. *Prostaglan. Leuk. Essential Fatty Acids*, **42**, 245–249.

HORROBIN, D.F. & MANKU, M. (1990). Clinical biochemistry of essential fatty acids. In *Omega-6 Essential Fatty Acids: Pathophysiology and Roles in Clinical Medicine*, Horrobin, D.F. (ed) pp. 21–35, Wiley-Liss, New York.

LEE, J.H. & SUGANO, M. (1986). Effects of linoleic and gamma-linolenic acid on 7, 12-dimethylbenz(a)anthracene-induced rat mammary tumours. *Nutr. Rep. Int.*, **34**, 1041–1049.

LEVI, S., GOODLAD, R.A., LEE, C.Y., STAMP, G., WALPORT, M.J., WRIGHT, N.A. & HODGSON, H.J.F. (1990). Inhibitory effect of non-steroidal anti-inflammatory drugs on mucosal cell proliferation associated with gastric ulcer healing. *Lancet*, **ii**, 840–843.

OVERGAARD, J., SAND HANSEN, H., ANDERSEN, A.P, HJELM-HANSEN, M., JORGENSEN, K., SANDBERG, E., BERTHELSEN, A., HAMMER, R.H. & PEDERSEN, M. (1989). Misonidazole combined with spilt-course radiotherapy in the treatment of invasive carcinoma of larynx and pharynx: report from the DAHANCA 2 study. *Int. J. Radiat. Oncol. Biol. Phys.*, **16**, 1065–1068.

OVERGAARD, J., SAND HANSEN, H., OVERGAARD, M., JORGENSEN, K., BASTHOLT, L., BERTHELSEN, A. & PEDERSEN, M. (1992). The Danish Head and Neck Cancer Study Group (DAHANCA) randomized trials with hypoxic radiosensitizers in carcinoma of the larynx and pharynx. In *Radiation Research, A Twentieth Century Perspective*, Dewey, W.C., Edington, M., Fry, R.J.M., Hall, E.J. & Whitmore, G.F. (eds) pp. 573–577, Academic Press; London.

ROBBINS, M.E.C. & HOPEWELL, J.W. (1986). Physiological factors effecting renal radiation tolerance: a guide to the treatment of late effects. *Br. J. Cancer*, **(Suppl VII)**, 265–267.

RUBIN, D.B., DRAB, E.A. & WARD, W.F. (1991). Physiological and biochemical markers of the endothelial cell response to irradiation. *Int. J. Radiat. Biol.*, **60**, 29–32.

SCHNEIDKRAUT, M.J., KOT, P.A., RAMWELL, P.N. & ROSE, J.C. (1984). Thromboxane and prostacyclin synthesis following whole body irradiation in rats. *J. Appl. Physiol: Respirat. Environ. Exercise Physiol.*, **57**, 833–838.

SINZINGER, H., CROMWELL, M. & FIRBAS, W. (1984). Long-lasting depression of rabbit aortic prostacyclin formation by single-dose irradiation. *Radiat. Res.*, **97**, 533–536.

STARK, G. (1991). The effect of ionising radiation on lipid membranes. *Biochim. Biophys. Acta*, **1071**, 103–122.

TOCHNER, Z., BARNES, M., MITCHELL, J.B., ORR, K., GLATSTEIN, E. & RUSSO, A. (1990). Protection by indomethacin against acute radiation esophagitis. *Digestion*, **47**, 81–87.

WARD, W.F., MOLTENI, A., TS'AO, C.H., KIM, Y.T. & HINZ, J.M. (1992). Radiation pneumotoxicity in rats: modification by inhibitors of angiotensin converting enzyme. *Int. J. Radiat. Oncol. Biol. Phys.*, **22**, 623–625.

WARD, W.F., MOLTENI, A., TS'AO, C. & HINZ, J.M. (1990). The effect of captopril on benign and malignant reactions in irradiated rat skin. *Br. J. Radiol.*, **63**, 349–354.

WESHLER, Z., RAZ, A., ROSENMANN, E., BIRAN, S., FUKS, Z. & ELDOR, A. (1987). Thromboxane and prostacyclin production by irradiated and perfused rat kidney. In *Prostaglandins and Lipid Metabolism in Radiation Injury*, Walden, T.L. & Hughes, H.N. (eds), pp. 219–224, Plenum: New York.

WILLIS, A.L. (1981). Nutritional and pharmacological factors in eicosanoid biology. *Nutr. Rev.*, **39**, 289–300.

ZURIER, R.B. (1990). Prostaglandin $E_1$: is it useful? *J. Rheumatol.*, **17**, 1439–1441.


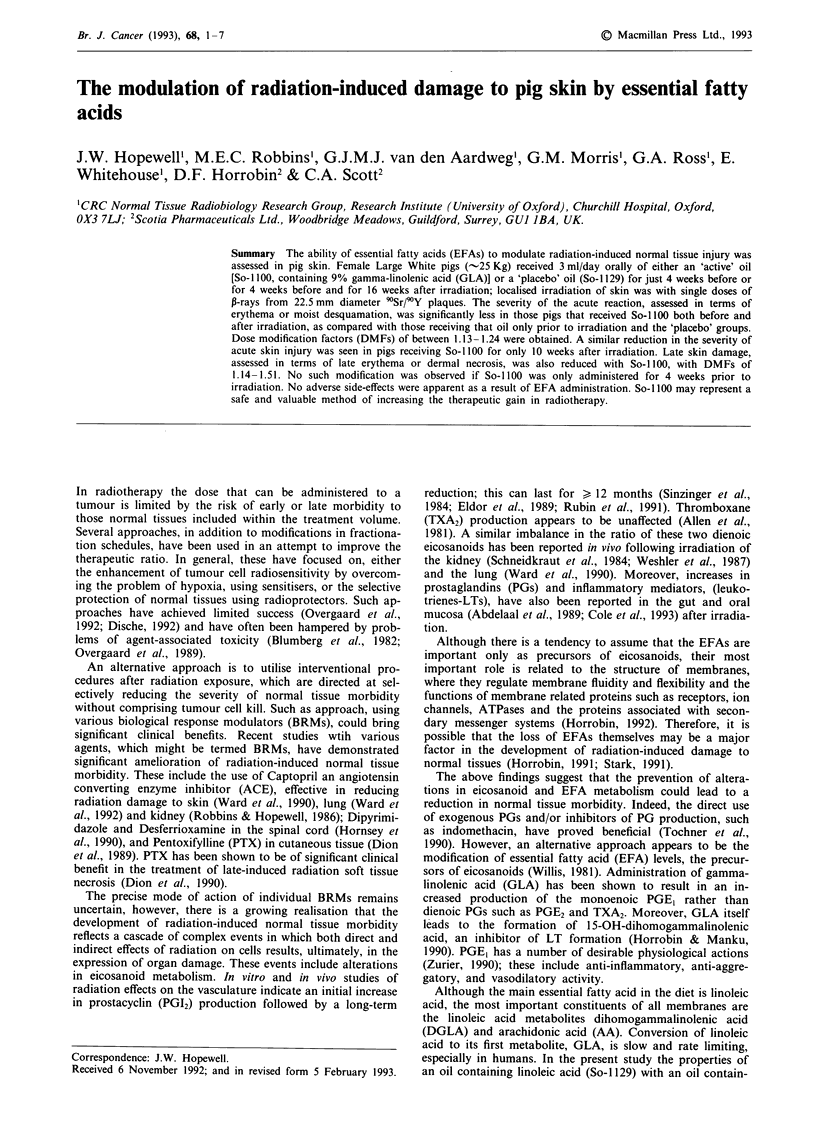

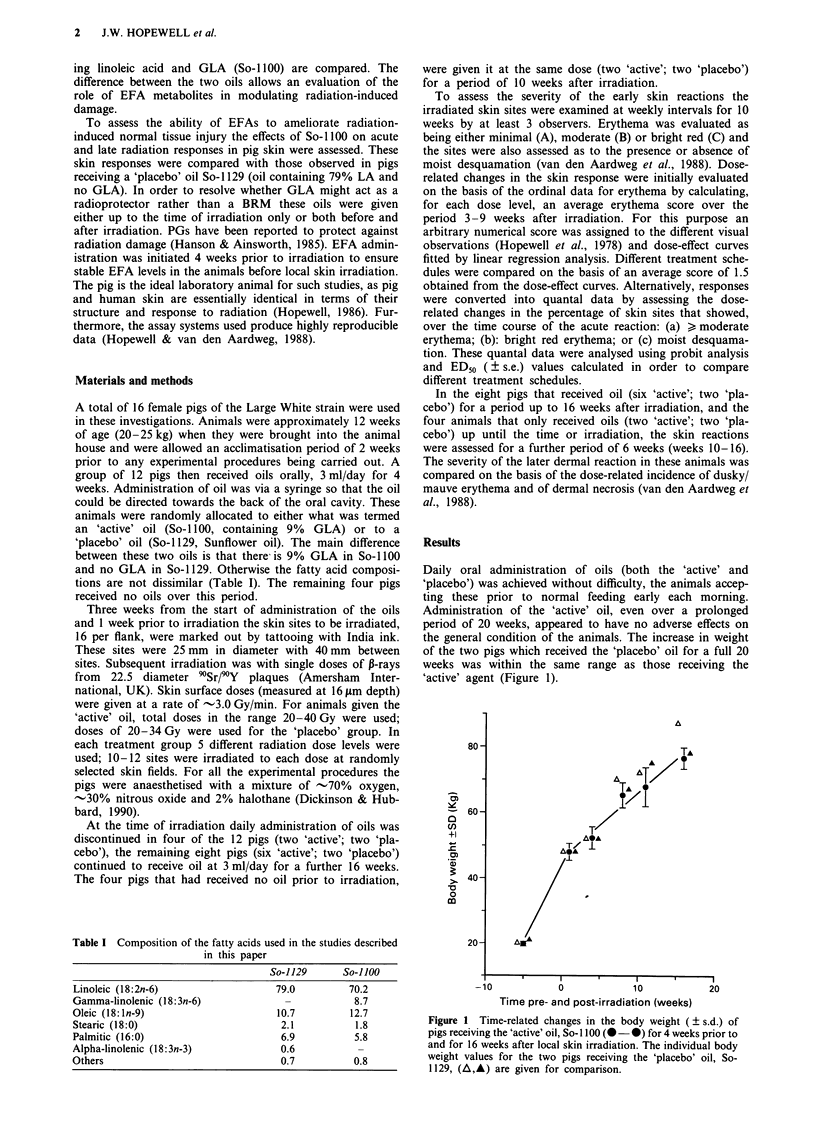

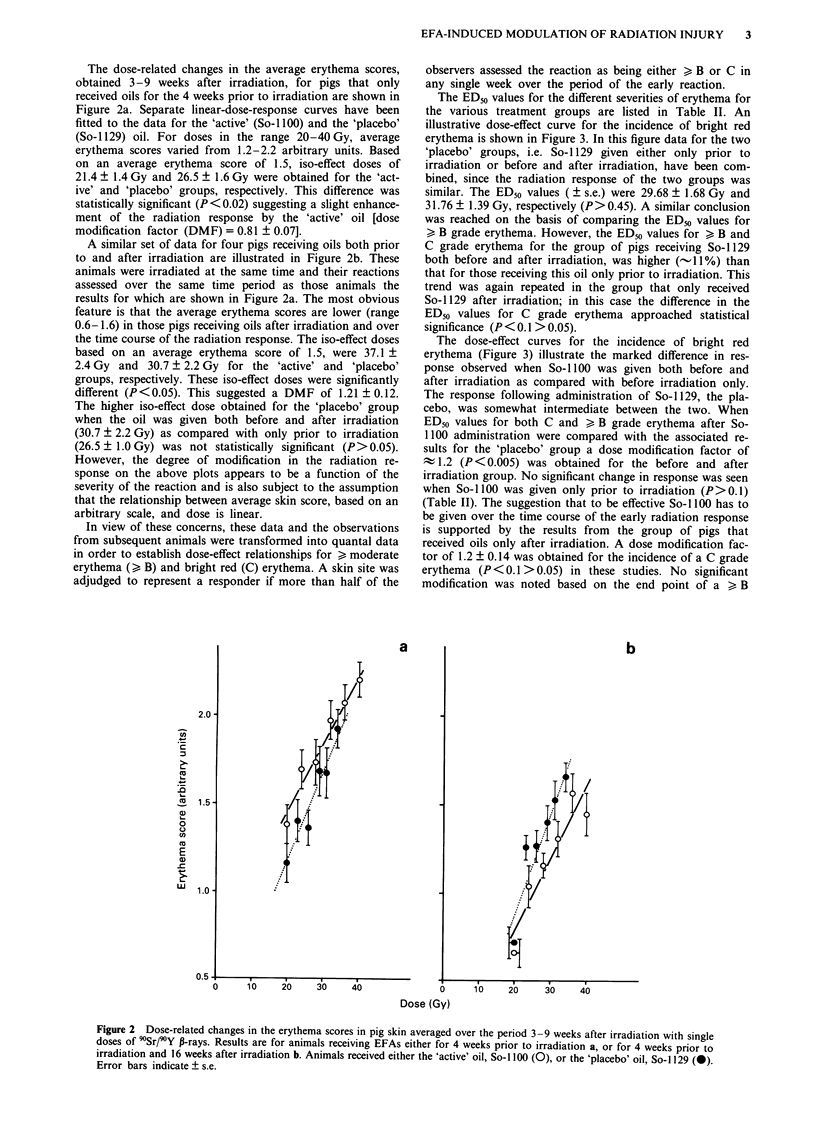

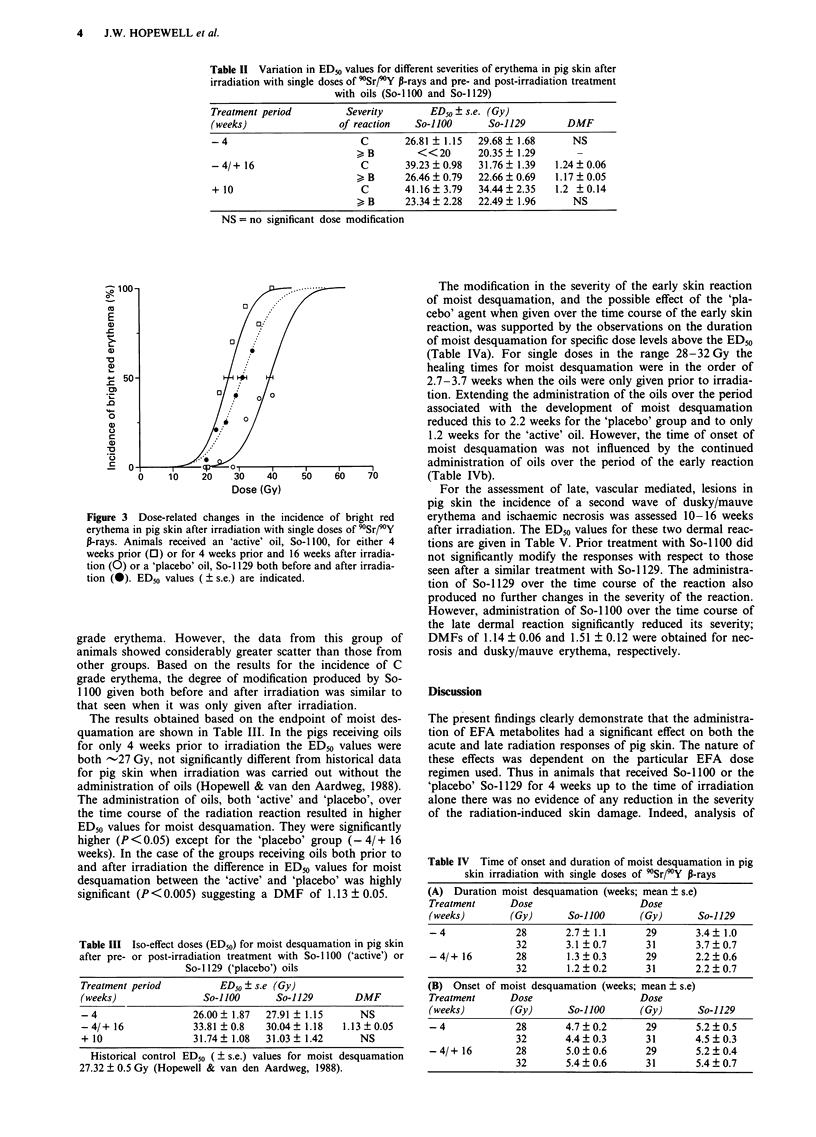

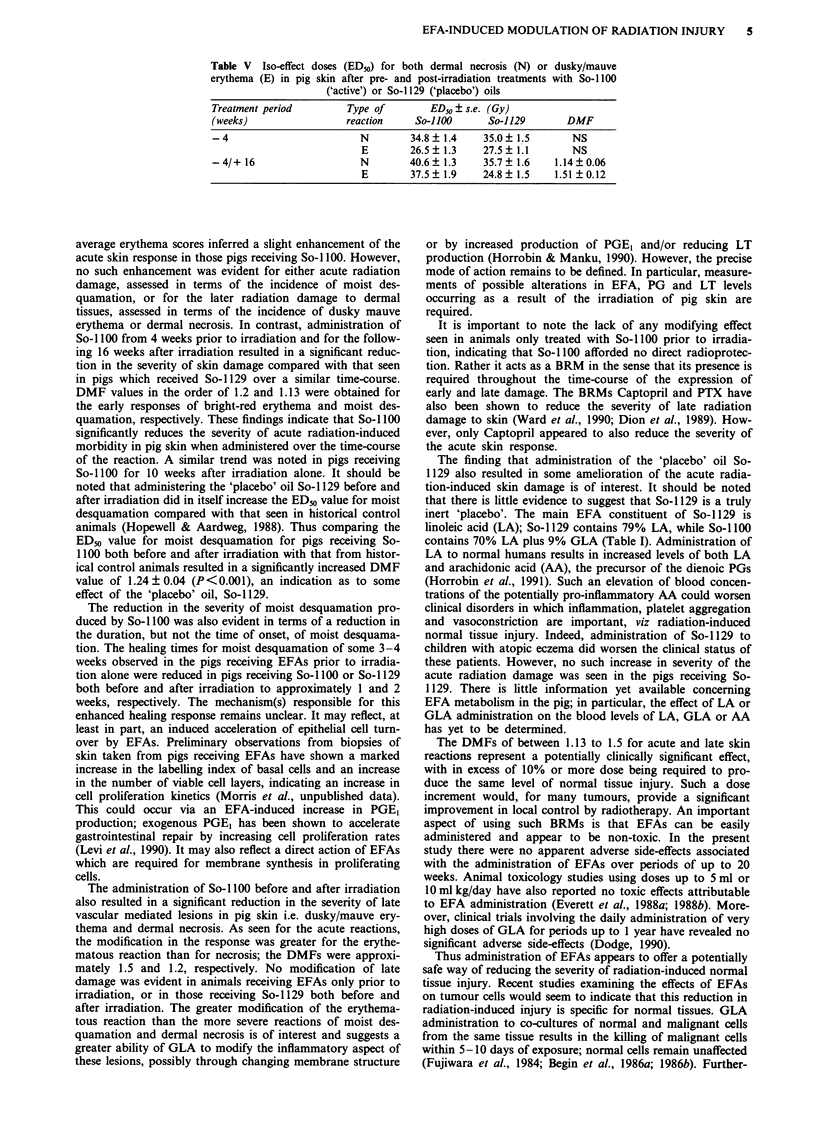

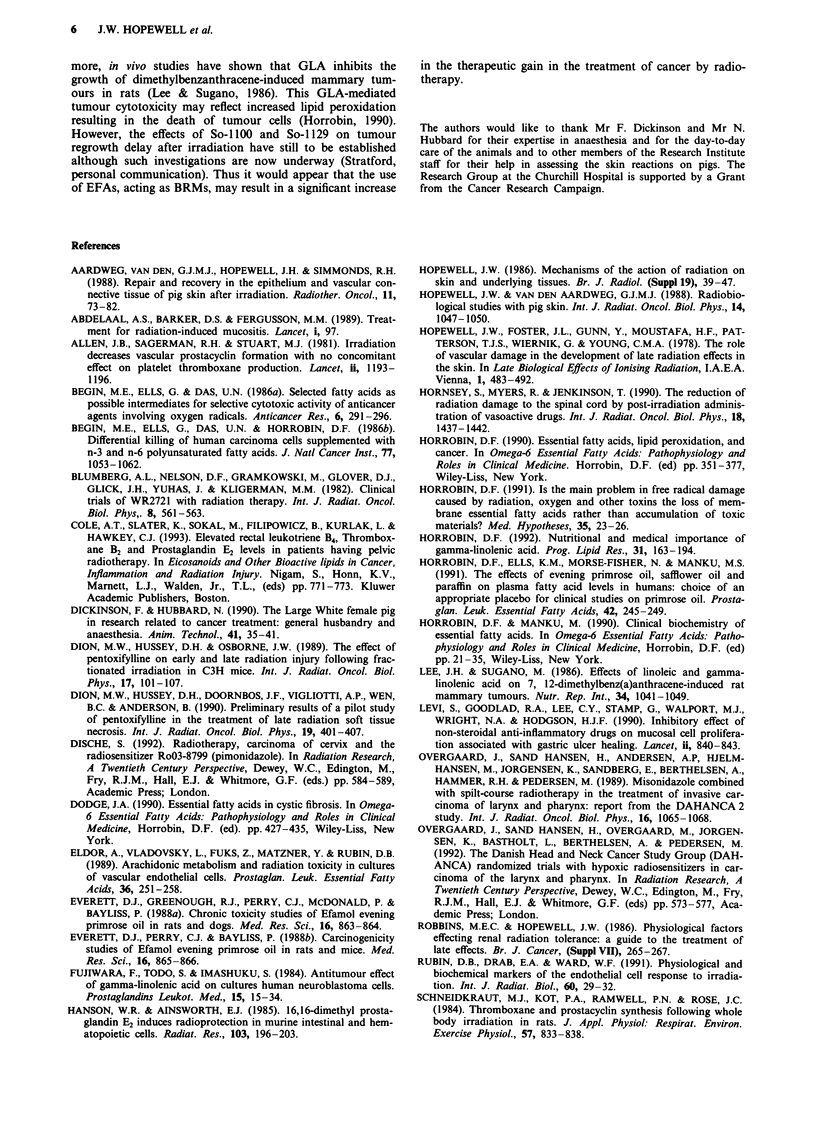

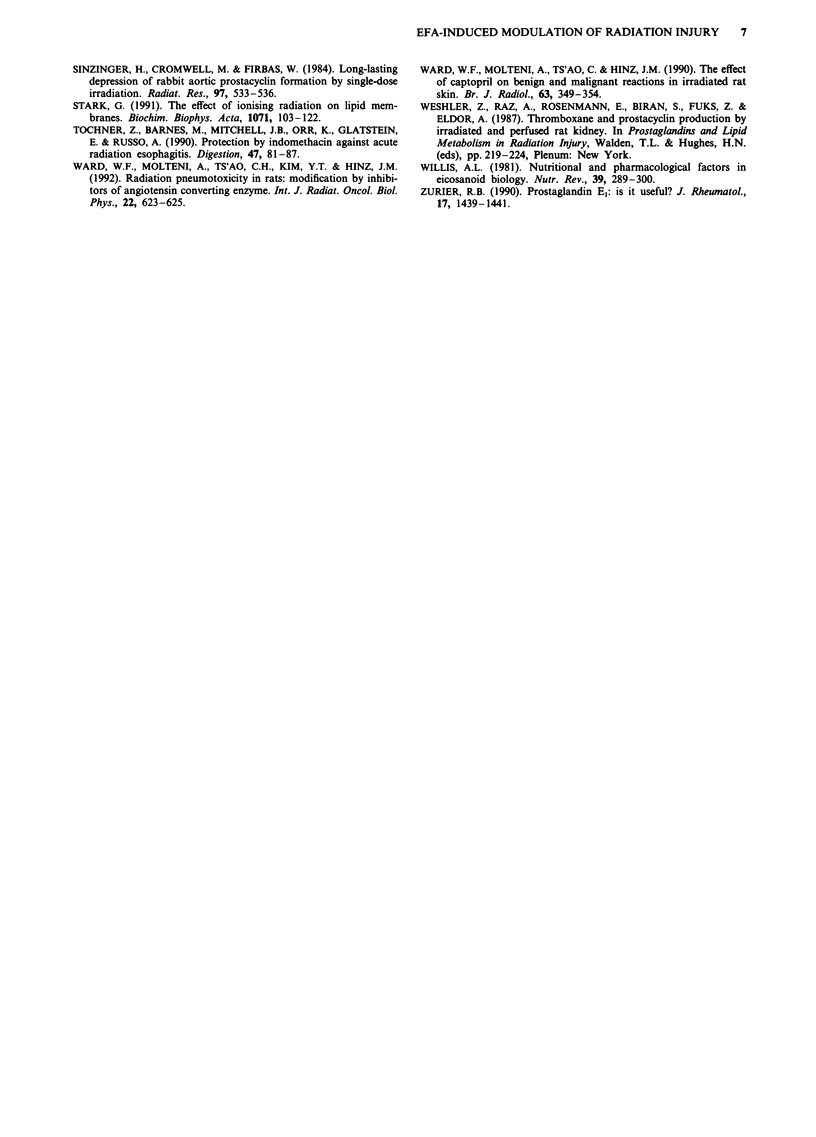


## References

[OCR_00774] Abdelaal A. S., Barker D. S., Fergusson M. M. (1989). Treatment for irradiation-induced mucositis.. Lancet.

[OCR_00778] Allen J. B., Sagerman R. H., Stuart M. J. (1981). Irradiation decreases vascular prostacyclin formation with no concomitant effect on platelet thromboxane production.. Lancet.

[OCR_00795] Blumberg A. L., Nelson D. F., Gramkowski M., Glover D., Glick J. H., Yuhas J. M., Kligerman M. M. (1982). Clinical trials of WR-2721 with radiation therapy.. Int J Radiat Oncol Biol Phys.

[OCR_00856] Booyens J., Engelbrecht P., le Roux S., Louwrens C. C., Van der Merwe C. F., Katzeff I. E. (1984). Some effects of the essential fatty acids linoleic acid and alpha-linolenic acid and of their metabolites gamma-linolenic acid, arachidonic acid, eicosapentaenoic acid, docosahexaenoic acid, and of prostaglandins A1 and E1 on the proliferation of human osteogenic sarcoma cells in culture.. Prostaglandins Leukot Med.

[OCR_00789] Bégin M. E., Ells G., Das U. N., Horrobin D. F. (1986). Differential killing of human carcinoma cells supplemented with n-3 and n-6 polyunsaturated fatty acids.. J Natl Cancer Inst.

[OCR_00784] Bégin M. E., Ells G., Das U. N. (1986). Selected fatty acids as possible intermediates for selective cytotoxic activity of anticancer agents involving oxygen radicals.. Anticancer Res.

[OCR_00821] Dion M. W., Hussey D. H., Doornbos J. F., Vigliotti A. P., Wen B. C., Anderson B. (1990). Preliminary results of a pilot study of pentoxifylline in the treatment of late radiation soft tissue necrosis.. Int J Radiat Oncol Biol Phys.

[OCR_00815] Dion M. W., Hussey D. H., Osborne J. W. (1989). The effect of pentoxifylline on early and late radiation injury following fractionated irradiation in C3H mice.. Int J Radiat Oncol Biol Phys.

[OCR_00840] Eldor A., Vlodavsky I., Fuks Z., Matzner Y., Rubin D. B. (1989). Arachidonic metabolism and radiation toxicity in cultures of vascular endothelial cells.. Prostaglandins Leukot Essent Fatty Acids.

[OCR_00861] Hanson W. R., Ainsworth E. J. (1985). 16,16-Dimethyl prostaglandin E2 induces radioprotection in murine intestinal and hematopoietic stem cells.. Radiat Res.

[OCR_00866] Hopewell J. W. (1986). Mechanisms of the action of radiation on skin and underlying tissues.. Br J Radiol Suppl.

[OCR_00870] Hopewell J. W., van den Aardweg G. J. (1988). Radiobiological studies with pig skin.. Int J Radiat Oncol Biol Phys.

[OCR_00882] Hornsey S., Myers R., Jenkinson T. (1990). The reduction of radiation damage to the spinal cord by post-irradiation administration of vasoactive drugs.. Int J Radiat Oncol Biol Phys.

[OCR_00904] Horrobin D. F., Ells K. M., Morse-Fisher N., Manku M. S. (1991). The effects of evening primrose oil, safflower oil and paraffin on plasma fatty acid levels in humans: choice of an appropriate placebo for clinical studies on primrose oil.. Prostaglandins Leukot Essent Fatty Acids.

[OCR_00894] Horrobin D. F. (1991). Is the main problem in free radical damage caused by radiation, oxygen and other toxins the loss of membrane essential fatty acids rather than the accumulation of toxic materials?. Med Hypotheses.

[OCR_00900] Horrobin D. F. (1992). Nutritional and medical importance of gamma-linolenic acid.. Prog Lipid Res.

[OCR_00922] Levi S., Goodlad R. A., Lee C. Y., Stamp G., Walport M. J., Wright N. A., Hodgson H. J. (1990). Inhibitory effect of non-steroidal anti-inflammatory drugs on mucosal cell proliferation associated with gastric ulcer healing.. Lancet.

[OCR_00930] Overgaard J., Hansen H. S., Andersen A. P., Hjelm-Hansen M., Jørgensen K., Sandberg E., Berthelsen A., Hammer R., Pedersen M. (1989). Misonidazole combined with split-course radiotherapy in the treatment of invasive carcinoma of larynx and pharynx: report from the DAHANCA 2 study.. Int J Radiat Oncol Biol Phys.

[OCR_00946] Robbins M. E., Hopewell J. W. (1986). Physiological factors effecting renal radiation tolerance: a guide to the treatment of late effects.. Br J Cancer Suppl.

[OCR_00951] Rubin D. B., Drab E. A., Ward W. F. (1991). Physiological and biochemical markers of the endothelial cell response to irradiation.. Int J Radiat Biol.

[OCR_00956] Schneidkraut M. J., Kot P. A., Ramwell P. W., Rose J. C. (1984). Thromboxane and prostacyclin synthesis following whole body irradiation in rats.. J Appl Physiol Respir Environ Exerc Physiol.

[OCR_00964] Sinzinger H., Cromwell M., Firbas W. (1984). Long-lasting depression of rabbit aortic prostacyclin formation by single-dose irradiation.. Radiat Res.

[OCR_00969] Stark G. (1991). The effect of ionizing radiation on lipid membranes.. Biochim Biophys Acta.

[OCR_00973] Tochner Z., Barnes M., Mitchell J. B., Orr K., Glatstein E., Russo A. (1990). Protection by indomethacin against acute radiation esophagitis.. Digestion.

[OCR_00980] Ward W. F., Molteni A., Ts'ao C. H., Kim Y. T., Hinz J. M. (1992). Radiation pneumotoxicity in rats: modification by inhibitors of angiotensin converting enzyme.. Int J Radiat Oncol Biol Phys.

[OCR_00984] Ward W. F., Molteni A., Ts'ao C., Hinz J. M. (1990). The effect of Captopril on benign and malignant reactions in irradiated rat skin.. Br J Radiol.

[OCR_00996] Willis A. L. (1981). Nutritional and pharmacological factors in eicosanoid biology.. Nutr Rev.

[OCR_01000] Zurier R. B. (1990). Prostaglandin E1: is it useful?. J Rheumatol.

[OCR_00768] van den Aardweg G. J., Hopewell J. W., Simmonds R. H. (1988). Repair and recovery in the epithelial and vascular connective tissues of pig skin after irradiation.. Radiother Oncol.

